# Mechanisms of Long Non-Coding RNAs in Biological Characteristics and Aerobic Glycolysis of Glioma

**DOI:** 10.3390/ijms222011197

**Published:** 2021-10-18

**Authors:** Ningning Zhao, Jiajie Zhang, Qian Zhao, Chao Chen, Huijuan Wang

**Affiliations:** College of Life Sciences, Northwest University, Xi’an 710069, China; 18729517815@163.com (N.Z.); qq476135834@163.com (J.Z.); xbdx1157019405@163.com (Q.Z.)

**Keywords:** glioma, lncRNAs, biological characteristics, aerobic glycolysis

## Abstract

Glioma is the most common and aggressive tumor of the central nervous system. The uncontrolled proliferation, cellular heterogeneity, and diffusive capacity of glioma cells contribute to a very poor prognosis of patients with high grade glioma. Compared to normal cells, cancer cells exhibit a higher rate of glucose uptake, which is accompanied with the metabolic switch from oxidative phosphorylation to aerobic glycolysis. The metabolic reprogramming of cancer cell supports excessive cell proliferation, which are frequently mediated by the activation of oncogenes or the perturbations of tumor suppressor genes. Recently, a growing body of evidence has started to reveal that long noncoding RNAs (lncRNAs) are implicated in a wide spectrum of biological processes in glioma, including malignant phenotypes and aerobic glycolysis. However, the mechanisms of diverse lncRNAs in the initiation and progression of gliomas remain to be fully unveiled. In this review, we summarized the diverse roles of lncRNAs in shaping the biological features and aerobic glycolysis of glioma. The thorough understanding of lncRNAs in glioma biology provides opportunities for developing diagnostic biomarkers and novel therapeutic strategies targeting gliomas.

## 1. Introduction

Glioma accounts for 30–40% of all primary tumors in the adult central nervous system (CNS). It can be categorized into four grades, of which glioblastoma multiforme (GMB) is the most aggressive form [1]. Patients with GBM have a median survival time of approximately 15 months, and the 5-year survival rate is about 5% [2]. Despite maximal surgical resection and subsequent radiochemotherapy, almost all patients suffer from tumor recurrence and drug resistance [3]. Thus, tremendous efforts have been invested in glioma research to find novel interventions and improve the treatment outcome. In the past decades, the genetic alterations, molecular mechanisms, and signaling pathways implicated in glioma have been extensively studied, and a large portion of this knowledge lays the foundations for developing novel therapeutic strategies. However, the complex biology in glioma cells, such as a high degree of heterogeneity, highly diffusive capacity, the immunosuppressive tumor microenvironment, and the development of drug resistance, frequently compromises the efficacy of existing treatments [4,5]. The exploration of new biomarkers for early diagnosis holds the promise to improve the effect of glioma treatment [6].

Uncontrolled cell proliferation is a hallmark of cancer cells which is accompanied by metabolic reprogramming, including the dysregulation of glucose catabolism and macromolecule biosynthesis [7]. Increased glycolysis and the reduced dependence on oxidative phosphorylation is a hallmark in cancer cells, which is known as the Warburg effect [8]. Unlike normal cells, even in the presence of abundant oxygen, cancer cells revert their glucose metabolism to glycolysis. This aerobic glycolysis is the direct or indirect result of oncogenic activation, which is believed to support the uncontrolled cell proliferation by providing precursors for nucleotide synthesis [9,10]. The Warburg effect contributes to the accelerated tumor growth by enhancing glucose uptake and lactate production in both the initial stage and the progression of various tumors [11]. Together, oncogene-induced glycolysis and multifaceted roles of glycolytic components underscore the significance of aerobic glycolysis in sustaining tumor progression. Hexokinase 2 (HK2), pyruvate kinaseM2 (PKM2), and lactate dehydrogenase A (LDHA) are key rate-limiting genes in glycolysis [12,13]. Targeting aerobic glycolysis is a potential strategy to selectively inhibit the growth of cancer cells, such as the usage of 2-Deoxy-d-Glucose and 3-Bromopyruvate [14,15]. In addition, the understanding of the dysregulation of glycolytic gene function and expression in cancer biology is crucial for developing targeted therapy.

Long noncoding RNAs (lncRNAs) are cataloged as noncoding RNAs whose length is longer than 200 nt [16]. Although lncRNAs have no or little protein-coding ability, they have been implicated in diverse physiological and pathological processes by modulating gene expression, including cell differentiation, chronical diseases, and cancers [17]. Accumulating evidence showed that lncRNAs also play roles in shaping the malignant phenotypes and rewiring the aerobic glycolysis of cancer [18,19]. A number of studies profiling the lncRNAs in cancerous tissues and para-cancerous normal tissues demonstrated that lncRNAs are dysregulated in a variety of human malignancies, including gastric cancer, bladder cancer, liver cancer, and brain cancers [20,21,22,23]. Aberrantly expressed lncRNAs have been implicated in glioma development through promoting cell proliferation and modulating aerobic glycolysis, and it has been proposed that dysregulated lncRNAs could serves as biomarkers for glioma diagnosis, prognostic prediction, and treatment targets [24,25]. However, the holistic landscape of lncRNAs in the regulation of biological characteristics and aerobic glycolysis of glioma remains to be completed. Here, we reviewed the current understanding of lncRNA expression and dysregulation in glioma and highlighted the roles of lncRNAs in modifying phenotypical features and rewiring the metabolism in glioma. We also discussed the challenges of the application of lncRNA as tumor biomarkers and therapeutic targets.

## 2. Overview of lncRNAs

**Figure 1:**
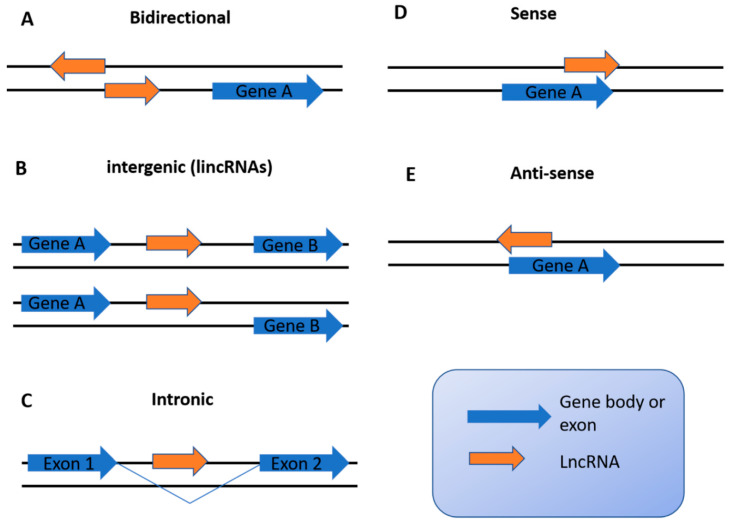
lncRNA classification according to their relative position to protein coding genes. Based on the genomic localization relative to protein-coding genes, lncRNAs can be classified into 5 categories: (**A**) bidirectional, (**B**) intergenic (lincRNAs), (**C**) intronic, (**D**) sense, and (**E**) antisense.

lncRNAs are defined as noncoding RNAs with more than 200 nt. They are transcribed by RNA polymerase II, which share structural features with mRNA, including a 5′-cap, splicing event, and a polyadenylated 3′ end (approximately 60% of lncRNAs have polyA tails) [26]. Compared with protein-coding mRNAs, the majority of lncRNAs are expressed at a relatively low level and exhibit tissue- or cell-type-specific expression [27,28]. Based on the genomic localization relative to protein-coding genes, lncRNAs can be classified into five categories: bidirectional, intergenic (lincRNAs), intronic, sense, and antisense (Figure 1) [28,29]. Bidirectional lncRNAs are the ones with transcription start site in close proximity to a protein-coding gene but are transcribed in the opposite direction. The distance between a bidirectional lncRNA and the promoter of the protein coding gene is less than 1 kb. The transcription of a bidirectional lncRNA often affects the expression of neighboring protein-coding genes, suggesting a cis-regulatory role of bidirectional lncRNAs [30]. LincRNAs are localized within the genomic interval of protein coding gene sequences. LincRNA transcripts differ from protein-coding transcripts with respect to length, number of exons per transcript, number of transcripts per gene, and transcriptional profile [31]. Intronic lncRNAs are derived from the introns within protein-coding exons. It is believed that intronic lncRNAs are spliced by pre-mRNA splicing machinery [32]. Sense and antisense lncRNAs are defined according to the nearest protein coding genes, and their transcript can partially or entirely overlap with one or more exons [33]. Sense lncRNAs overlap with protein-coding exons and are transcribed in the same direction, while antisense lncRNAs are transcribed in the opposite direction of the overlapping genes.

**Figure 2:**
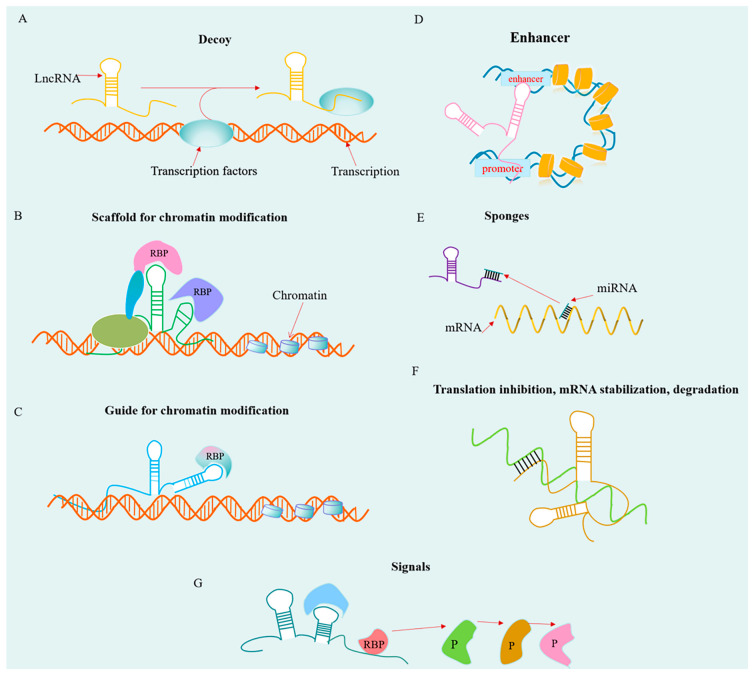
The molecular mechanisms of lncRNAs regulating cellular processes. (**A**) lncRNAs can act as decoys to facilitate transcription; (**B**,**C**) lncRNAs can seve as a scaffold or guide for chromatin modification; (**D**) lncRNAs promote R-loop formation to facilitate transcription; (**E**) lncRNAs can seve as ceRNAs by sponging to miRNA; (**F**) lncRNAs can regulate mRNA translation, stability, and degradation; (**G**) Signals—the RBP association with lncRNAs can contribute to conformational alterations that activate signal molecules.

Genome-wide sequencing revealed that lncRNAs are localized in both the nucleus and cytoplasm, indicating diversified functional roles [34]. In the nucleus, lncRNAs are frequently associated with chromatin, regulating chromatin organization, and gene expression, while a large portion of lncRNAs are exported into the cytoplasm, which can interact with RNA-binding proteins (RBPs), mRNAs, and other non-coding RNAs [34,35]. The molecular mechanisms by which lncRNAs regulate cellular processes depend on their subcellular location (Figure 2). lncRNAs in the nucleus were reported to regulate chromatin structures and gene expression through DNA binding or acting as the scaffold for the chromatin-remodeling complex. Jain et al. showed that LncPRESS1 serves as a decoy for SIRT6 in modulating gene expression of ECS [36]. Chen et al. found that lncRNA NEAT1 is located in the nucleus and promotes GBM progression by regulating the activity of the WNT/b-catenin pathway and acting as a scaffold of the polycomb complex protein EZH2 [37]. Li et al. demonstrated that HOTAIR act as a guide to localize PRC2 in glioma progression [38]. Xiang et al. reported that lncRNA CCAT1-L locates to 515 kb upstream of the MYC-515 site and is capable of chromatin looping between the MYC-515 promoter and its enhancers in colorectal cancer (CRC) [39]. In the cytoplasm, lncRNAs can affect the activity of miRNA by severing as a sponge to dampen the target factors (competing endogenous RNAs (ceRNAs) mechanism) and regulate mRNA translation, stability, and protein function. Zhang et al. demonstrated that lncRNA ENST00000413528 acts as a sponge of microRNA-593-5p to regulate glioma growth through targeting polo-like kinase 1 [40]. Yoon et al. found that LincRNA-p21 can inhibit target mRNAs JUNB and CTNNB1 translation in Hela cells [41]. Zhang et al. demonstrated that lncRNA ARLNC1 regulates the stabilization of AR transcript through RNA-RNA interaction in prostate cancer cells [42]. Wang et al. revealed that LINC01426 facilitates glioma progression via the PI3K/AKT signaling pathway [43]. A growing body of evidence continues to expand the biological roles of lncRNAs in a multitude of physiological and pathological conditions, adding another layer of complexity in epigenetic control by non-coding RNAs.

## 3. Roles of lncRNAs in Biological Characteristics of Glioma

**Figure 3:**
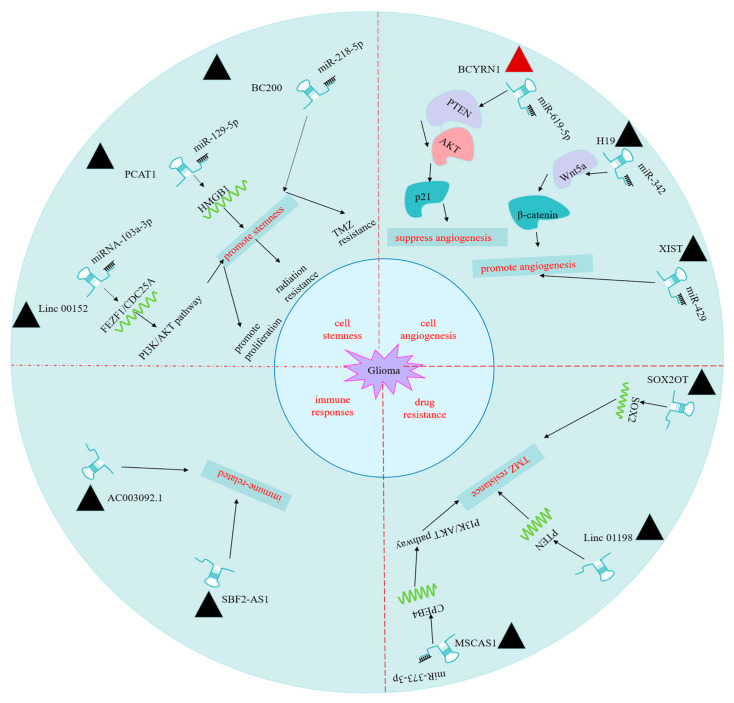
Biological function of some important lncRNAs in glioma. 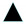 represents the oncogene, 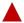 represents the tumor suppressor gene.

Recent studies demonstrated that, compared with nontumoral brain tissues, a large number of lncRNAs are aberrantly expressed in glioma [44,45]. In addition, the differential expression of lncRNAs is widely implicated in the occurrence, metastasis, and drug resistance of gliomas [45,46]. In the following sections, the biological functions of well-characterized lncRNAs in glioma are summarized (Figure 3).

### 3.1. lncRNAs Regulate Glioma Cells Stemness

Glioma stem cells (GSCs) are a population of poorly differentiated and highly heterogeneous cells with stem cell properties. GSC_S_ have the ability to self-renew and differentiate into other subtypes of glioma cells and are characterized by the increased resistance to chemotherapy and radiotherapy, which may account for the failure of treatment, high recurrence rate, and poor prognosis in glioma [47]. Su et al. reported that lncRNA BC200 could regulate miR-218-5p expression and promote glioma stemness, which leads to resistance to Temozolomide (TMZ) in glioma cells [48]. Zhang et al. revealed that the lncRNA PCAT1 plays a crucial role in radiation sensitivity by modulating the miR-129-5p/HMGB1 axis. Knocking down lncRNA PCAT1 in GSCs promotes radiation sensitivity and increases the efficacy of radiotherapy [49]. Another study demonstrated that Linc00152 augments the malignant phenotype of GSCs through the miR-103a-3p/FEZF1/CDC25A pathway, indicating that targeting Linc00152 could be used as a potential therapeutic approach [50]. Since the GSC subpopulation has been recognized as a key contributing factor in the resistance to conventional therapies as well as glioma recurrence, targeted elimination of GSCs may enhance the treatment outcome of the existing therapies.

### 3.2. lncRNAs Regulate Angiogenesis of Glioma Cells

Angiogenesis plays a vital role in the proliferation and migration of glioma cells, which is also a hallmark in glioblastoma [51]. In order to enhance the blood flow to meet the nutrient and oxygen demands of tumor growth, tumor cells generally secrete angiogenic factors, such as angiogenin, matrix metalloproteinases (MMPs), and vascular endothelial growth factors (VEGFs), to stimulate local neovascularization [52]. In addition, a hypoxic condition not only affects the metabolism in tumor microenvironment, it also regulates angiogenesis. Accumulating evidence has highlighted the roles of lncRNAs in angiogenesis under hypoxia [53,54]. Mu et al. recently reported that lncRNA BCYRN1 suppresses glioma tumorigenesis by inactivating the PTEN/AKT/p21 pathway via targeting miR-619-5p [55]. In contrast, Zhou et al. found that lncRNA H9 promotes cell proliferation, migration, and angiogenesis of glioma by targeting the miR-342/Wnt5a/β-catenin signaling axis [56]. Another study revealed that lncRNA XIST facilitates glioma tumorigenesis and angiogenesis by acting as a sponge for miR-429 [57]. It seems that different lncRNAs could indirectly mediate the progression of gliomas by targeting different miRNAs, which in turn regulates the downstream target proteins involved in angiogenesis and tumorigenesis.

### 3.3. lncRNAs Contribute to Glioma Drug Resistance

A standard treatment after maximal surgical resection of glioma tissue involves the combined radiotherapy and chemotherapy [58]. However, nearly all glioma patients will eventually develop drug resistance and suffer from tumor recurrence [59]. In addition to the roles in glioma stem cells, accumulating evidence starts to unveil the crucial roles of lncRNAs in drug resistance development. TMZ is the standard oral alkylating agent for chemotherapy in glioma, which can pass the blood–brain barrier (BBB), whereas TMZ resistance is primarily responsible for the poor efficacy of glioma chemotherapy [3,60]. Various lncRNAs are associated with tumor chemoresistance, including glioma, liver, prostate, and cervical cancers [61,62], and lncRNAs have also been implicated in TMZ resistance [45,63]. Liu et al. reported that lncRNA SOX2OT enhances the resistance to TMZ by promoting SOX2 expression and inhibiting apoptosis [64]. Chen et al. demonstrated that LINC01198 overexpression promotes TMZ resistance by elevating NEDD4-1-dependent suppression of oncogene PTEN [65]. Another study showed that lncRNA MSC-AS1 knockdown inhibits TMZ resistance of glioma through modulating the miR-373-3p/CPEB4 axis and activating the PI3K/Akt signaling pathway [66]. Since resistance towards a single agent can be easily mounted by different lncRNAs, combinatory therapies using multiple drugs may limit the chance of drug resistance and enhance the treatment outcome. In addition, exosomal lncRNAs can cross the BBB and can be detected in blood, urine, saliva, breast milk, and cerebrospinal fluid (CSF). Therefore, exosomal lncRNAs will have a great potential in novel biomarkers and therapeutics of glioma [67].

### 3.4. lncRNAs May Be Implicated in Immune Responses in Glioma

The tumor microenvironment is characterized by a milieu of immunosuppressive cells, hindering antitumor immune responses [68]. Patients with malignant glioma display multiple immune deficiency, including CD4 lymphopenia, increased fraction of regulatory T cells (Tregs) in peripheral blood, infiltration of immunosuppressive macrophages, and the impaired Th1 cytokine production and cell-mediated immunity [4,69]. Several recent studies profiling lncRNAs in glioma identified immune-related signatures of lncRNA expression [70,71,72], which shows predictive power in the glioma pathogenesis and clinical prognosis. For instance, lncRNA AC003092.1 was demonstrated to be associated with glioma-immunosuppressive microenvironment through analyzing The Cancer Genome Atlas (TCGA) database [73]. lncRNA SBF2-AS1 is associated with immunity in lower-grade glioma (LGG) through analyzing TCGA and the Chinese Glioma Genome Atlas (CGGA) database [74]. In addition, lncRNAs are involved in the regulation of inflammatory gene expression, which can shape the immune microenvironment. For example, lncRNA NEAT1 regulates interleukin IL-8 transcription and affects the activity of different immune cells [75]. LincRNA-Cox2 modulates inflammatory genes in macrophages by targeting the chromatin remodeling activity of the SWI/SNF complex in the nucleus [76]. The complex interplay between lncRNAs and the immune microenvironment in glioma remain to be further investigated, and the functional characterization of immune-related lncRNAs in glioma can shed lights on the immunosuppressive mechanisms and the development of targeted immunotherapy.

## 4. Potential Clinical Application of lncRNAs in Glioma

With the rapid advancement of high-throughput sequencing technology, the number of dysregulated lncRNAs identified in glioma continues to rise [77,78]. Differential expression of lncRNAs is correlated to tumor grade, prognosis, and drug resistance of glioma cells [79,80]. An increasing number of lncRNAs have been characterized with the potentials of clinical applications in glioma. In the following section, we discussed the clinical application potentials of lncRNAs in glioma (summarized in Table 1).

### 4.1. lncRNAs as Promising Biomarkers for Glioma Diagnosis

The power of lncRNAs as biomarkers for glioma diagnosis is beginning to emerge. A team of researchers evaluated lncRNA HOTAIR expression in peripheral serum of 43 patients with GBM, 23 LGG (low-grade glioma), and 40 healthy controls [81]. Their results showed that lncRNA HOTAIR expression level in GMB patients was significantly higher than that of LGG patients, and lncRNA HOTAIR level in all brain tumor patients was also significantly higher than that of healthy controls. This finding suggests that lncRNA HOTAIR is a promising biomarker for glioma patients as well as for evaluating the aggressiveness of glioma. lncRNA HOTAIR was estimated to have a sensitivity of 86.1% and specificity of 87.5% in predicting glioma [81]. Another study recruited 42 glioma patients and 10 healthy controls, and applied microarray chips to profile dysregulated lncRNAs in tumor tissues and tumor-adjacent normal tissues [82]. They found that lncRNA miR210HG expression was significantly upregulated in tumor tissues compared to tumor-adjacent normal tissues of glioma patients. A higher serum miR210HG level was also observed in glioma patients as compared to healthy controls [82]. Mu et al. performed RNA-seq to uncover the expression profiling of lncRNAs in glioma tissue samples, and they found that lncRNA BCYRN1 serves as a tumor suppressor via inactivating the CUEDC2/PTEN/AKT/p21 pathway [55]. With more sequencing datasets becoming available, an increasing number of lncRNAs have been associated with the tumorigenesis and malignant degree of gliomas. The identification of novel lncRNAs not only improves our understanding of mechanisms underlying the progression of glioma, but also holds the potential for them to serve as novel biomarkers for diagnosis. It is anticipated that the use of a panel of multiple lncRNAs specifically dysregulated in glioma patients will generate more reliable diagnoses.

### 4.2. lncRNAs as Biomarkers for Glioma Prognosis

Recent studies also start to elucidate that some lncRNAs are strongly associated with glioma progression and malignant phenotypes. For example, Song et al. analyzed lncRNA signatures and clinical pathological data of glioma patients in TCGA. 510 LGG and 160 GBM RNA-seq data from TCGA were retrieved. They found that the prognosis of high histology grade glioma was much poorer, and four lncRNAs (AC064875.2, HOTAIRM1, LINC00908, and RP11-84A19.3) were highly correlated with both high neoplasm grade and worse prognosis [84]. Interestingly, these lncRNAs seem to be involved in neutrophil-mediated immunity and cell adhesion junction [83], implying that they may affect the immunity in the glioma tumor microenvironment. The study by Wang et al. discovered that the expression level of LINC01503 in glioma tissues and cells was significantly upregulated and was associated with a higher tumor grade and a poorer clinical prognosis [84]. They further revealed that LINC01503 could regulate Wnt/β-catenin signaling to promote glioma progression, thus predicting worse prognosis. Another recent study revealed that exosomes derived from hypoxic glioma stem cells contain an elevated level of Linc01060, which supports glioma progression by regulating the MZF1/c-Myc/HIF-1α axis and is correlated with a poor clinical prognosis [85]. It is noteworthy that recent studies highlighted the power of lncRNA expression profiling and the usage of multiple dysregulated lncRNAs as the risk signatures to predict the therapeutic efficacy and prognosis [86,87].

### 4.3. lncRNAs as Therapeutic Targets

As the dysregulation of lncRNAs mediates a multifaceted biology in glioma initiation and progression, targeting lncRNAs is a potential approach to restore the dysregulated biological processes. Using small interfering RNA (siRNA) or antisense oligonucleotides to target tumor-specific lncRNAs is a promising strategy under intensive research, although the clinical trials are very limited [88]. Using CRISPRi-based genome-wide screening, a recent study established a generalizable approach to rapidly identify novel therapeutic targets [89]. They also found that targeting lncGRS-1 by antisense oligonucleotides suppressed glioma tumor growth in 3D culture and sensitized glioma cells to radiotherapy. Huang et al. recently reported that lncRNA GAS5-AS1 expression was correlated with the glioma tumor grade and the overexpression of GAS5-AS1 suppressed glioma tumor growth in nude mice [90]. lncRNA GAS5-AS1 suppresses glioma proliferation by targeting the miR-106b-5p/TUSC2 axis, suggesting GAS5-AS1 overexpression as a novel therapeutic approach. Liu et al. reported that lncRNA SNHG1 was highly expressed in glioma and facilitated glioma progression, and silencing lncRNA SNHG1 inhibited glioma progression in vitro and in vivo [91]. Sheng et al. confirmed that lncRNA ST7-AS1 inhibits the progression of glioma through a p53/ST7-AS1/PTBP1 feedback loop, which might be a potential therapeutic target for glioma treatment [92]. It is worth mentioning that the activities of some lncRNAs seem to be conserved across multiple cancer types; thereby, these lncRNAs may serve as potential pan-cancer therapeutic targets. For example, lncRNA PVT1 is overexpressed in various malignant tumors, including glioma, HCC, gastric cancer, and NSCLC, which correlates with a poorer prognosis [93,94]. In addition, other lncRNAs, such as H19, MALAT1, HOTAIR, and NEAT1, are also conserved across multiple cancer types and could serve as potential therapeutic targets [88,95,96].

## 5. lncRNAs Modulate Aerobic Glycolysis of Glioma

**Figure 4:**
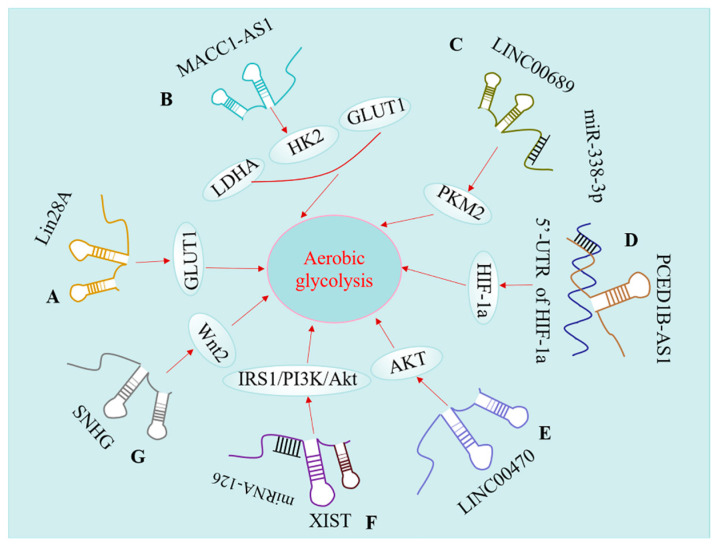
lncRNAs modulate the aerobic glycolysis of glioma. (**A**) Lin28 can enhance the expression of GLUT1; (**B**) MACC1-AS1 can promote the expression of LDHA, HK2, and GLUT1; (**C**) LINC00689 can increase PKM2 expression by binding to miR-338-3p; (**D**) PCED1B activates HIF-1a by binding to 5′-UTR of HIF-1a mRNA; (**E**) LINC00470 activates the AKT pathway to promote aerobic glycolysis of glioma; (**F**) XIST regulates the glucose metabolism via targeting the IRS1/PI3K/Akt pathway in glioma. (**G**) SNHG can promote cell growth and aerobic glycolysis by upregulating Wnt2 in GBM.

Recent studies demonstrated that numerous lncRNAs play critical roles in cancer metabolism. Understanding the relationship between lncRNAs and aerobic glycolysis can provide new strategies for cancer treatment [13,14,15]. More and more lncRNAs have been functionally characterized in aerobic glycolysis of glioma, suggesting that lncRNAs modulate the aerobic glycolysis of glioma (Figure 4).

### 5.1. lncRNAs Regulate GLUT Levels in Glioma

Glucose is the main source of energy for cells, which is imported into cells through glucose transporters (GLUTs) and broken down by glycolysis and the TCA cycle to generate cellular energy [97]. Glioma cells tend to upregulate GLUT1 and GLUT3 to accelerate the uptake of glucose [98,99]. Recent studies revealed that lncRNAs can regulate the expression of GLUT genes [100]. Lu et al. reported that the expression of Lin28A in GBM was upregulated, and the knockdown of Lin28A suppressed glycolysis and proliferation in glioma cells by indirectly reducing the transcription of PKM2 (pyruvate kinase muscle 2) and GLUT1 [101]. Interestingly, GLUT3 isoform seems to be the predominant glucose transporter in highly malignant glial cells of human brain, suggesting a functional diversity of GLUT1 and GLUT3 in the progression of glioma [102].

### 5.2. lncRNAs Modulate Key Glycolytic Enzyme

Hexokinase (HK) is recognized as the first vital enzyme of the glycolytic pathway, which can promote the uptake of glucose into glioma cells via GLUTs by glucose phosphorylation reaction [103]. Numerous studies reported the upregulation of hexokinase isoform 2 (HK2) in cancer cells and its association with the overall survival of patients with glioma [104,105,106]. Zheng et al. investigated the mechanism of lncRNA MACC1-AS1-mediated progression of glioma. The abnormal upregulation of lncRNA MACC1-AS1 in A172 and U251 glioma cells seems closely associated with glucose metabolism. MACC1-AS1 overexpression significantly enhances the level of HK2 and GLUT1 [107], suggesting that MACC1-AS1 promotes glycolysis and leads to metabolic reprogramming in glioma progression. Recent studies discovered that other glycolytic enzymes, such as PKM2 and lactate dehydrogenase A (LDHA), are also upregulated in glioma [108,109]. lncRNAs are implicated in the regulation of key glycolytic enzymes in various cancers, such as liver cancer, colorectal cancer, and non-small-cell lung cancer (NSCLC) [110,111]. Wang et al. revealed that lncRNA HULC accelerates the aerobic glycolysis of HepG2 cells via upregulating LDHA and PKM2 [112]. Bian et al. discovered that lncRNA-FEZF1-AS1 regulates the glycolysis of colorectal cancer through direct interaction with PKM2 to facilitate tumor proliferation and metastasis [113]. Liu et al. reported that lncRNA LINC00689 is highly expressed in glioma relative to normal brain tissues. They demonstrated that the overexpression of LINC00689 can promote glycolysis of glioma cells by binding to miR-338-3p and increasing PKM2 expression [114]. Nevertheless, the mechanisms by which lncRNAs rewire the metabolic program to favor aerobic glycolysis in glioma remains to be fully elucidated.

### 5.3. lncRNA Regulates Glucose Metabolism by Targeting Different Signaling Pathways

#### 5.3.1. HIF-1a Pathway

Hypoxia is a key feature of the tumor microenvironment in solid tumors, and hypoxia-inducible factor 1 (HIF-1) is frequently activated under hypoxia [115]. HIF-1 is an important mediator of the Warburg effect, which comprises of the O_2_-responsive subunit HIF-1a and a constitutively expressed subunit HIF-1b. The HIF-1 transcription complex is able to modulate the expression of a multitude of genes through binding to hypoxia response elements [116]. Previous studies revealed that HIF-1a plays significant roles in the regulation of aerobic glycolysis. HIF-1a predominantly promotes glycolysis by three manners: (1) upregulating GLUT1 expression and contributing to elevated glucose uptake; (2) modulating glycolytic enzymes to increase glucose consumption; (3) upregulating LDHA, which facilitates pyruvate usage and microenvironment acidification [117,118]. Yao et al. discovered that overexpression of lncRNA PCED1B-AS1 enhances glucose uptake in GBM cells. PCED1B-AS1 binds directly to the 5′-UTR of HIF-1a mRNA, increasing the protein level of HIF-1a by potentiating HIF-1a mRNA translation, thereby promoting the Warburg effect and tumorigenesis [119]. The regulation of the HIF-1a pathway not only induces metabolic reprogramming, but also contributes to angiogenesis [120]. Not surprisingly, the activation of hypoxia response can in turn regulate the expression of lncRNAs [121,122].

#### 5.3.2. Phosphoinositide 3-Kinase (PI3K)/Akt Pathway

The PI3K/Akt/mTOR pathway is an extensively explored intracellular pathway in various tumors. PI3K can be activated via extracellular stimuli, such as platelet-derived growth factor (PDGF). Xiao et al. reported that PDGF facilitates the Warburg effect by activating the PI3K signaling pathway [123]. The activation of PI3K results in the phosphorylation of two important residues on AKT kinase, which initiates a downstream phosphorylation cascade. PI3Ks are classified into three subtypes based on the structure and substrate specificity [124]. Class I PI3Ks promote the formation of the second messenger phosphatidylinositol-3,4,5-trisphosphate (PIP3) from phosphatidylinositol-4,5-bisphosphate (PIP2). Lipid phosphatase PTEN negatively regulates the PI3K singling pathway by converting PIP3 to PIP2. PIP3 not only binds to the pleckstrin homology domain of Akt at the plasma membrane, but also activates phosphoinositide-dependent protein kinase 1 to phosphorylate Akt at Thr308 [125]. The complete activation of AKT occurs by mTOR-dependent phosphorylation on serine 473 (S473) [126]. The activation of the PI3K/Akt/mTOR pathway promotes the Warburg effect mainly by three main mechanisms: (1) enhancing the expression level of GLUTs to augment glucose uptake; (2) upregulating key glycolytic enzymes, such as PKM2, HK2, and LDHA; (3) AKT activation contributing to an increased HIF-1a activity [127,128].

Recent studies start to unveil the functional roles of lncRNAs in the regulation of the PI3K/Akt/mTOR pathway in various tumors [129]. Liu et al. demonstrated that lncRNA LINC00470 inhibits the ubiquitination of HK1 through activating AKT, which contributes to the augmented glycolysis of GBM [130]. A study by Cheng et al. revealed that lncRNA-XIST modulates the glucose metabolism by targeting the IRS1/PI3K/Akt pathway in glioma [131]. lncRNA-XIST seems to act as a competing endogenous RNA of miR-126, and lncRNA-XIST knockdown reduces the tumorigenicity of glioblastoma cells. Since the PI3K/Akt pathway is a key contributor to cell survival and drug resistance [132], these lncRNAs may also contribute to drug resistance development in glioma.

#### 5.3.3. Wnt Pathway

The Wnt signaling pathway is a multifactorial pathway implicated in a myriad of pathophysiological processes such as embryonic development, cell migration, cell proliferation, metastasis, and drug resistance in cancers [133,134]. Canonical WNT pathway activation leads to the stabilization and accumulation of β-catenin, and its nuclear translocation leads to transcriptional changes of target genes. The Wnt signaling pathway is aberrantly activated in cancer cells and contributes to the changes in cellular metabolism [135]. Activated Wnt signaling promotes the up-regulation of MCT-1, CYC1, and ATP synthase, resulting in enhanced production of lactate in aerobic glycolysis [126]. Wnt signaling activation also upregulates glycolysis-related genes such as GLUT-1, LDH, PKM2, and other c-Myc dependent genes to promote glycolysis, nucleotide, and fatty acid synthesis in tumor cells [136,137]. Zhang et al. reported that lncRNA SNHG promotes cell growth and aerobic glycolysis in GBM through upregulating Wnt2 [138]. In addition, an increasing number of studies have investigated the interplay between lncRNAs and Wnt/β-catenin signaling in glioma. Several lncRNAs, such as NEAT1, CCAT264, CCND2-AS265, DANCER66, and AB07361463, have been implicated in tumor proliferation, migration, and invasion in glioma via targeting the Wnt/β-catenin signaling pathway [37,139,140,141,142]. A recent study revealed that lncRNA NEAT1 is an oncogene in glioblastoma, which promotes cell growth and invasion by increasing β-catenin nuclear transport [37]. lncRNA CCAT2 is also upregulated in glioma tissues, and its knockdown inhibits cell proliferation and tumorigenesis possibly by suppressing the downstream genes of the Wnt/β-catenin signaling pathway [139]. Although in these studies, the effects of lncRNAs on cellular metabolism and aerobic glycolysis were not investigated, the well-established role of Wnt pathways in cancer metabolism [143] indicates that lncRNAs targeting the Wnt pathway may also contribute to the metabolic reprograming in glioma.

## 6. Conclusions and Future Perspectives

Despite the advancement of cancer treatment, glioma, particularly GBM, remains as one of the most aggressive cancers. A growing body of studies have revealed that a large number of lncRNAs are implicated in different aspects of glioma biology, such as cell proliferation, angiogenesis, stemness, drug resistance, and metabolic reprogramming. Certain lncRNAs might be useful biomarkers for the diagnosis, prognosis, and drug response prediction in glioma. However, the complex interplay among lncRNAs, mRNAs, proteins, and other non-coding RNAs has not been fully elucidated. Most studies of lncRNAs in glioma usually focus on ceRNA modulatory networks. The mechanisms by which lncRNAs are dysregulated in glioma remain largely unknown. A holistic picture of lncRNA expression regulation and the molecular targets will provide useful information to understand the complex biology of glioma progression.

A high rate of aerobic glycolysis is a key characteristic of cancer cells, which promotes uncontrolled proliferation. The key glycolytic enzymes, including GLUT-1, LDHA, and PKM2, are frequently upregulated in cancers and are promising metabolic targets for treatment. At present, the roles of lncRNAs in cell metabolism regulation have been well established. Those studies provide novel insights into the connection between lncRNAs and the Warburg effect in glioma. The identification and characterization of glycolysis-related lncRNAs offers potential novel intervention targeting the metabolic reprogramming.

The development of lncRNA-based therapy in glioma faces several challenges. Drug delivery across the intact BBB is a key consideration in therapeutic formulation for glioma. At present, due to the poor stability and impaired drug uptake, therapeutic strategies targeting lncRNAs still remain limited. Novel delivery systems such as exosome and nanoparticles may remarkably enhance their bioavailability and stability [144]. Moreover, since only a small fraction of lncRNAs has been well characterized, future studies are required to fully delineate the intricate mechanisms by which lncRNAs regulate metabolic reprogramming in glioma. With increasing efforts to be invested in the research of lncRNAs in glioma, we anticipate that lncRNAs will become a key player in the diagnosis, prognosis, prediction, and drug development in glioma.

## Figures and Tables

**Table 1:** lncRNAs with diagnostic, prognosis, and therapeutic potential in glioma.

Expression Level of lncRNAs	lncRNAs	Mechanism	Reported Potential Clinical Application	References
Upregulated	HOTAIR		Diagnosis and prognosis	[81]
Upregulated	miR210HG		Diagnosis	[82]
Downregulated	BCYRN1	Sponge miR-619-5p and interact with the CUEDC2/PTEN/AKT/p21 pathway	Diagnosis, therapeutic target, and prognosis	[55]
Upregulated	AC064875.2		Neoplasm grade and prognosis	[83]
Upregulated	HOTAIRM1		Neoplasm grade and prognosis	[83]
Upregulated	LINC00908		Neoplasm grade and prognosis	[83]
Upregulated	RP11-84A19.3		Neoplasm grade and prognosis	[83]
Upregulated	LINC 01503	Interact with Wnt/β-catenin pathway	Tumorigenesis, progression, and prognosis	[84]
Upregulated	LINC 01060	Interact with MZF1/c-Myc/HIF-1α axis	Prognosis and progression	[85]
Downregulated	TBX5-AS1		therapeutic target and prognosis	[86]
Upregulated	LNC01545		therapeutic target and prognosis	[86]
Upregulated	WDR11-AS1		therapeutic target and prognosis	[86]
Upregulated	NDUFA6-DT		therapeutic target and prognosis	[86]
Upregulated	FRY-AS1		therapeutic target and prognosis	[86]
Upregulated	H19		prognosis	[87]
Downregulated	HAR1A		prognosis	[87]
Upregulated	NEAT1		prognosis	[88]
Upregulated	lncGRS-1		Therapeutic target	[89]
Downregulated	GAS5-AS1	Interact with miR-106b-5p/TUSC2 axis	Proliferation, therapeutic target	[90]
Upregulated	SNHG1		Progression, therapeutic target	[91]
Downregulated	ST7-AS1	Interact with p53/ST7-AS1/PTBP1 feedback loop	Therapeutic target	[92]
Upregulated	PVT1		Pan-cancer therapeutic target and prognosis	[87,93]

## Data Availability

Not applicable.

## References

[1] Santosh V., Sravya P., Gupta T., Muzumdar D., Chacko G., Suri V., Epari S., Balasubramaniam A., Radotra B.D., Chatterjee S. (2019). ISNO consensus guidelines for practical adaptation of the WHO 2016 classification of adult diffuse gliomas. Neurol. India.

[2] Tamimi A.F., Juweid M., De Vleeschouwer S. (2017). Epidemiology and Outcome of Glioblastoma. Glioblastoma.

[3] Osuka S., Van Meir E.G. (2017). Overcoming therapeutic resistance in glioblastoma: The way forward. J. Clin. Investig..

[4] DeCordova S., Shastri A., Tsolaki A.G., Yasmin H., Klein L., Singh S.K., Kishore U. (2020). Molecular Heterogeneity and Immunosuppressive Microenvironment in Glioblastoma. Front. Immunol..

[5] Nicholson J.G., Fine H.A. (2021). Diffuse Glioma Heterogeneity and Its Therapeutic Implications. Cancer Discov..

[6] Kan L.K., Drummond K., Hunn M., Williams D., O’Brien T.J., Monif M. (2020). Potential biomarkers and challenges in glioma diagnosis, therapy and prognosis. BMJ Neurol. Open.

[7] Phan L.M., Yeung S.C., Lee M.H. (2014). Cancer metabolic reprogramming: Importance, main features, and potentials for precise targeted anti-cancer therapies. Cancer Biol. Med..

[8] Zheng J. (2012). Energy metabolism of cancer: Glycolysis versus oxidative phosphorylation (Review). Oncol. Lett..

[9] Martinez-Outschoorn U.E., Lin Z., Ko Y.H., Goldberg A.F., Flomenberg N., Wang C., Pavlides S., Pestell R.G., Howell A., Sotgia F. (2011). Understanding the metabolic basis of drug resistance: Therapeutic induction of the Warburg effect kills cancer cells. Cell Cycle.

[10] Vander Heiden M.G., Cantley L.C., Thompson C.B. (2009). Understanding the Warburg effect: The metabolic requirements of cell proliferation. Science.

[11] San-Millan I., Brooks G.A. (2017). Reexamining cancer metabolism: Lactate production for carcinogenesis could be the purpose and explanation of the Warburg Effect. Carcinogenesis.

[12] Patel M.S., Mahmood S., Jung J., Rideout T.C. (2021). Reprogramming of aerobic glycolysis in non-transformed mouse liver with pyruvate dehydrogenase complex deficiency. Physiol. Rep..

[13] Ji L., Shen W., Zhang F., Qian J., Jiang J., Weng L., Tan J., Li L., Chen Y., Cheng H. (2021). Worenine reverses the Warburg effect and inhibits colon cancer cell growth by negatively regulating HIF-1alpha. Cell Mol. Biol. Lett..

[14] Pajak B., Siwiak E., Soltyka M., Priebe A., Zielinski R., Fokt I., Ziemniak M., Jaskiewicz A., Borowski R., Domoradzki T. (2019). 2-Deoxy-d-Glucose and Its Analogs: From Diagnostic to Therapeutic Agents. Int. J. Mol. Sci..

[15] Fan T., Sun G., Sun X., Zhao L., Zhong R., Peng Y. (2019). Tumor Energy Metabolism and Potential of 3-Bromopyruvate as an Inhibitor of Aerobic Glycolysis: Implications in Tumor Treatment. Cancers.

[16] Kung J.T., Colognori D., Lee J.T. (2013). Long noncoding RNAs: Past, present, and future. Genetics.

[17] Li G., Deng L., Huang N., Sun F. (2021). The Biological Roles of lncRNAs and Future Prospects in Clinical Application. Diseases.

[18] Lin W., Zhou Q., Wang C.Q., Zhu L., Bi C., Zhang S., Wang X., Jin H. (2020). lncRNAs regulate metabolism in cancer. Int. J. Biol. Sci..

[19] Pan Y.F., Feng L., Zhang X.Q., Song L.J., Liang H.X., Li Z.Q., Tao F.B. (2011). Role of long non-coding RNAs in gene regulation and oncogenesis. Chin. Med. J..

[20] Foroughi K., Amini M., Atashi A., Mahmoodzadeh H., Hamann U., Manoochehri M. (2018). Tissue-Specific Down-Regulation of the Long Non-Coding RNAs PCAT18 and LINC01133 in Gastric Cancer Development. Int. J. Mol. Sci..

[21] Cao Y.P., Zhou J., Li W.J., Shao Y., Zheng S.Y., Tian T., Xie K.P., Yan X. (2018). Long Non-Coding RNA Expression Profiles for the Characterization of Different Bladder Cancer Grade. Cell Physiol. Biochem..

[22] Yadav B., Pal S., Rubstov Y., Goel A., Garg M., Pavlyukov M., Pandey A.K. (2021). lncRNAs associated with glioblastoma: From transcriptional noise to novel regulators with a promising role in therapeutics. Mol. Nucleic Acids.

[23] Wang B., Yang S., Zhao W. (2020). Long Non-Coding RNA NRAD1 and LINC00152 are Highly Expressed and Associated with Prognosis in Patients with Hepatocellular Carcinoma. Oncol. Targets.

[24] Stackhouse C.T., Gillespie G.Y., Willey C.D. (2020). Exploring the Roles of lncRNAs in GBM Pathophysiology and Their Therapeutic Potential. Cells.

[25] Kiran M., Chatrath A., Tang X., Keenan D.M., Dutta A. (2019). A Prognostic Signature for Lower Grade Gliomas Based on Expression of Long Non-Coding RNAs. Mol. Neurobiol..

[26] Sun Q., Hao Q., Prasanth K.V. (2018). Nuclear Long Noncoding RNAs: Key Regulators of Gene Expression. Trends Genet..

[27] Liu S.J., Nowakowski T.J., Pollen A.A., Lui J.H., Horlbeck M.A., Attenello F.J., He D., Weissman J.S., Kriegstein A.R., Diaz A.A. (2016). Single-cell analysis of long non-coding RNAs in the developing human neocortex. Genome Biol..

[28] Waseem M., Liu Y., Xia R. (2020). Long Non-Coding RNAs, the Dark Matter: An Emerging Regulatory Component in Plants. Int. J. Mol. Sci..

[29] Ma L., Bajic V.B., Zhang Z. (2013). On the classification of long non-coding RNAs. RNA Biol..

[30] Wei W., Pelechano V., Jarvelin A.I., Steinmetz L.M. (2011). Functional consequences of bidirectional promoters. Trends Genet..

[31] Ulitsky I., Bartel D.P. (2013). lincRNAs: Genomics, evolution, and mechanisms. Cell.

[32] Krchnakova Z., Thakur P.K., Krausova M., Bieberstein N., Haberman N., Muller-McNicoll M., Stanek D. (2019). Splicing of long non-coding RNAs primarily depends on polypyrimidine tract and 5′ splice-site sequences due to weak interactions with SR proteins. Nucleic Acids Res..

[33] Latge G., Poulet C., Bours V., Josse C., Jerusalem G. (2018). Natural Antisense Transcripts: Molecular Mechanisms and Implications in Breast Cancers. Int. J. Mol. Sci..

[34] Statello L., Guo C.J., Chen L.L., Huarte M. (2021). Gene regulation by long non-coding RNAs and its biological functions. Nat. Rev. Mol. Cell Biol..

[35] Fernandes J.C.R., Acuna S.M., Aoki J.I., Floeter-Winter L.M., Muxel S.M. (2019). Long Non-Coding RNAs in the Regulation of Gene Expression: Physiology and Disease. Noncoding RNA.

[36] Jain A.K., Xi Y., McCarthy R., Allton K., Akdemir K.C., Patel L.R., Aronow B., Lin C., Li W., Yang L. (2016). LncPRESS1 Is a p53-Regulated lncRNA that Safeguards Pluripotency by Disrupting SIRT6-Mediated De-acetylation of Histone H3K56. Mol. Cell.

[37] Chen Q., Cai J., Wang Q., Wang Y., Liu M., Yang J., Zhou J., Kang C., Li M., Jiang C. (2018). Long Noncoding RNA NEAT1, Regulated by the EGFR Pathway, Contributes to Glioblastoma Progression Through the WNT/beta-Catenin Pathway by Scaffolding EZH2. Clin. Cancer Res..

[38] Li Y., Ren Y., Wang Y., Tan Y., Wang Q., Cai J., Zhou J., Yang C., Zhao K., Yi K. (2019). A Compound AC1Q3QWB Selectively Disrupts HOTAIR-Mediated Recruitment of PRC2 and Enhances Cancer Therapy of DZNep. Theranostics.

[39] Xiang J.F., Yin Q.F., Chen T., Zhang Y., Zhang X.O., Wu Z., Zhang S., Wang H.B., Ge J., Lu X. (2014). Human colorectal cancer-specific CCAT1-L lncRNA regulates long-range chromatin interactions at the MYC locus. Cell Res..

[40] Zhang R., Wei R.L., Du W., Zhang L.W., Du T., Geng Y.D., Wei X.T. (2019). Long noncoding RNA ENST00000413528 sponges microRNA-593-5p to modulate human glioma growth via polo-like kinase 1. CNS Neurosci..

[41] Yoon J.H., Abdelmohsen K., Srikantan S., Yang X., Martindale J.L., De S., Huarte M., Zhan M., Becker K.G., Gorospe M. (2012). LincRNA-p21 suppresses target mRNA translation. Mol. Cell.

[42] Zhang Y., Pitchiaya S., Cieslik M., Niknafs Y.S., Tien J.C., Hosono Y., Iyer M.K., Yazdani S., Subramaniam S., Shukla S.K. (2018). Analysis of the androgen receptor-regulated lncRNA landscape identifies a role for ARLNC1 in prostate cancer progression. Nat. Genet..

[43] Wang S.J., Wang H., Zhao C.D., Li R. (2018). Long noncoding RNA LINC01426 promotes glioma progression through PI3K/AKT signaling pathway and serves as a prognostic biomarker. Eur. Rev. Med. Pharmacol. Sci..

[44] Chen G., Cao Y., Zhang L., Ma H., Shen C., Zhao J. (2017). Analysis of long non-coding RNA expression profiles identifies novel lncRNA biomarkers in the tumorigenesis and malignant progression of gliomas. Oncotarget.

[45] Zottel A., Samec N., Videtic Paska A., Jovcevska I. (2020). Coding of Glioblastoma Progression and Therapy Resistance through Long Noncoding RNAs. Cancers.

[46] Chen X., Guo G., Lu Y., Wang S., Zhang Y., Huang Q. (2021). Mechanisms and functions of long noncoding RNAs in glioma (Review). Oncol. Rep..

[47] Bao S., Wu Q., McLendon R.E., Hao Y., Shi Q., Hjelmeland A.B., Dewhirst M.W., Bigner D.D., Rich J.N. (2006). Glioma stem cells promote radioresistance by preferential activation of the DNA damage response. Nature.

[48] Su Y.K., Lin J.W., Shih J.W., Chuang H.Y., Fong I.H., Yeh C.T., Lin C.M. (2020). Targeting BC200/miR218-5p Signaling Axis for Overcoming Temozolomide Resistance and Suppressing Glioma Stemness. Cells.

[49] Zhang P., Liu Y., Fu C., Wang C., Duan X., Zou W., Zhao T. (2019). Knockdown of long non-coding RNA PCAT1 in glioma stem cells promotes radiation sensitivity. Med. Mol. Morphol..

[50] Yu M., Xue Y., Zheng J., Liu X., Yu H., Liu L., Li Z., Liu Y. (2017). Linc00152 promotes malignant progression of glioma stem cells by regulating miR-103a-3p/FEZF1/CDC25A pathway. Mol. Cancer.

[51] Ahir B.K., Engelhard H.H., Lakka S.S. (2020). Tumor Development and Angiogenesis in Adult Brain Tumor: Glioblastoma. Mol. Neurobiol..

[52] Quintero-Fabian S., Arreola R., Becerril-Villanueva E., Torres-Romero J.C., Arana-Argaez V., Lara-Riegos J., Ramirez-Camacho M.A., Alvarez-Sanchez M.E. (2019). Role of Matrix Metalloproteinases in Angiogenesis and Cancer. Front. Oncol..

[53] Chen L., Endler A., Shibasaki F. (2009). Hypoxia and angiogenesis: Regulation of hypoxia-inducible factors via novel binding factors. Exp. Mol. Med..

[54] Zhu X., Pan H., Liu L. (2021). Long noncoding RNA network: Novel insight into hepatocellular carcinoma metastasis (Review). Int. J. Mol. Med..

[55] Mu M., Niu W., Zhang X., Hu S., Niu C. (2020). lncRNA BCYRN1 inhibits glioma tumorigenesis by competitively binding with miR-619-5p to regulate CUEDC2 expression and the PTEN/AKT/p21 pathway. Oncogene.

[56] Zhou Q., Liu Z.Z., Wu H., Kuang W.L. (2020). lncRNA H19 Promotes Cell Proliferation, Migration, and Angiogenesis of Glioma by Regulating Wnt5a/beta-Catenin Pathway via Targeting miR-342. Cell Mol. Neurobiol..

[57] Cheng Z., Li Z., Ma K., Li X., Tian N., Duan J., Xiao X., Wang Y. (2017). Long Non-coding RNA XIST Promotes Glioma Tumorigenicity and Angiogenesis by Acting as a Molecular Sponge of miR-429. J. Cancer.

[58] Fernandes C., Costa A., Osorio L., Lago R.C., Linhares P., Carvalho B., Caeiro C., De Vleeschouwer S. (2017). Current Standards of Care in Glioblastoma Therapy. Glioblastoma.

[59] Shergalis A., Bankhead A., Luesakul U., Muangsin N., Neamati N. (2018). Current Challenges and Opportunities in Treating Glioblastoma. Pharmacol. Rev..

[60] Lee S.Y. (2016). Temozolomide resistance in glioblastoma multiforme. Genes Dis..

[61] Ding L., Wang R., Shen D., Cheng S., Wang H., Lu Z., Zheng Q., Wang L., Xia L., Li G. (2021). Role of noncoding RNA in drug resistance of prostate cancer. Cell Death Dis..

[62] Chi Y., Wang D., Wang J., Yu W., Yang J. (2019). Long Non-Coding RNA in the Pathogenesis of Cancers. Cells.

[63] Zeng H., Xu N., Liu Y., Liu B., Yang Z., Fu Z., Lian C., Guo H. (2017). Genomic profiling of long non-coding RNA and mRNA expression associated with acquired temozolomide resistance in glioblastoma cells. Int. J. Oncol..

[64] Liu B., Zhou J., Wang C., Chi Y., Wei Q., Fu Z., Lian C., Huang Q., Liao C., Yang Z. (2020). lncRNA SOX2OT promotes temozolomide resistance by elevating SOX2 expression via ALKBH5-mediated epigenetic regulation in glioblastoma. Cell Death Dis..

[65] Chen W.L., Chen H.J., Hou G.Q., Zhang X.H., Ge J.W. (2019). LINC01198 promotes proliferation and temozolomide resistance in a NEDD4-1-dependent manner, repressing PTEN expression in glioma. Aging.

[66] Li C., Feng S., Chen L. (2021). MSC-AS1 knockdown inhibits cell growth and temozolomide resistance by regulating miR-373-3p/CPEB4 axis in glioma through PI3K/Akt pathway. Mol. Cell Biochem..

[67] Cheng J., Meng J., Zhu L., Peng Y. (2020). Exosomal noncoding RNAs in Glioma: Biological functions and potential clinical applications. Mol. Cancer.

[68] Labani-Motlagh A., Ashja-Mahdavi M., Loskog A. (2020). The Tumor Microenvironment: A Milieu Hindering and Obstructing Antitumor Immune Responses. Front. Immunol..

[69] Vega E.A., Graner M.W., Sampson J.H. (2008). Combating immunosuppression in glioma. Future Oncol..

[70] Wang X., Gao M., Ye J., Jiang Q., Yang Q., Zhang C., Wang S., Zhang J., Wang L., Wu J. (2020). An Immune Gene-Related Five-lncRNA Signature for to Predict Glioma Prognosis. Front. Genet..

[71] Li Y., Jiang T., Zhou W., Li J., Li X., Wang Q., Jin X., Yin J., Chen L., Zhang Y. (2020). Pan-cancer characterization of immune-related lncRNAs identifies potential oncogenic biomarkers. Nat. Commun..

[72] Xia P., Li Q., Wu G., Huang Y. (2021). An Immune-Related lncRNA Signature to Predict Survival in Glioma Patients. Cell Mol. Neurobiol..

[73] Guo X.Y., Zhong S., Wang Z.N., Xie T., Duan H., Zhang J.Y., Zhang G.H., Liang L., Cui R., Hu H.R. (2021). Immunogenomic Profiling Demonstrate AC003092.1 as an Immune-Related eRNA in Glioblastoma Multiforme. Front. Genet..

[74] Zhang Q., Liu X.J., Li Y., Ying X.W., Chen L. (2021). Prognostic Value of Immune-Related lncRNA SBF2-AS1 in Diffuse Lower-Grade Glioma. Technol. Cancer Res. Treat..

[75] Zhang P., Cao L., Zhou R., Yang X., Wu M. (2019). The lncRNA Neat1 promotes activation of inflammasomes in macrophages. Nat. Commun..

[76] Hu G., Gong A.Y., Wang Y., Ma S., Chen X., Chen J., Su C.J., Shibata A., Strauss-Soukup J.K., Drescher K.M. (2016). LincRNA-Cox2 Promotes Late Inflammatory Gene Transcription in Macrophages through Modulating SWI/SNF-Mediated Chromatin Remodeling. J. Immunol..

[77] Huang Y.K., Yu J.C. (2015). Circulating microRNAs and long non-coding RNAs in gastric cancer diagnosis: An update and review. World J. Gastroenterol..

[78] Pan Y.B., Zhu Y., Zhang Q.W., Zhang C.H., Shao A., Zhang J. (2020). Prognostic and Predictive Value of a Long Non-coding RNA Signature in Glioma: A lncRNA Expression Analysis. Front. Oncol..

[79] Rezaei O., Tamizkar K.H., Sharifi G., Taheri M., Ghafouri-Fard S. (2020). Emerging Role of Long Non-Coding RNAs in the Pathobiology of Glioblastoma. Front. Oncol..

[80] Mahinfar P., Baradaran B., Davoudian S., Vahidian F., Cho W.C., Mansoori B. (2021). Long Non-Coding RNAs in Multidrug Resistance of Glioblastoma. Genes.

[81] Tan S.K., Pastori C., Penas C., Komotar R.J., Ivan M.E., Wahlestedt C., Ayad N.G. (2018). Serum long noncoding RNA HOTAIR as a novel diagnostic and prognostic biomarker in glioblastoma multiforme. Mol. Cancer.

[82] Min W., Dai D., Wang J., Zhang D., Zhang Y., Han G., Zhang L., Chen C., Li X., Li Y. (2016). Long Noncoding RNA miR210HG as a Potential Biomarker for the Diagnosis of Glioma. PLoS ONE.

[83] Song L., Zhang S., Duan C., Ma S., Hussain S., Wei L., Chu M. (2019). Genome-wide identification of lncRNAs as novel prognosis biomarkers of glioma. J. Cell Biochem..

[84] Wang H., Sheng Z.G., Dai L.Z. (2019). Long non-coding RNA LINC01503 predicts worse prognosis in glioma and promotes tumorigenesis and progression through activation of Wnt/beta-catenin signaling. Eur. Rev. Med. Pharmacol. Sci..

[85] Li J., Liao T., Liu H., Yuan H., Ouyang T., Wang J., Chai S., Li J., Chen J., Li X. (2021). Hypoxic Glioma Stem Cell-Derived Exosomes Containing Linc01060 Promote Progression of Glioma by Regulating the MZF1/c-Myc/HIF1alpha Axis. Cancer Res..

[86] Niu X., Sun J., Meng L., Fang T., Zhang T., Jiang J., Li H. (2020). A Five-lncRNAs Signature-Derived Risk Score Based on TCGA and CGGA for Glioblastoma: Potential Prospects for Treatment Evaluation and Prognostic Prediction. Front. Oncol..

[87] Chen Y., Guo Y., Chen H., Ma F. (2020). Long Non-coding RNA Expression Profiling Identifies a Four-Long Non-coding RNA Prognostic Signature for Isocitrate Dehydrogenase Mutant Glioma. Front. Neurol..

[88] Arun G., Diermeier S.D., Spector D.L. (2018). Therapeutic Targeting of Long Non-Coding RNAs in Cancer. Trends Mol. Med..

[89] Liu S.J., Malatesta M., Lien B.V., Saha P., Thombare S.S., Hong S.J., Pedraza L., Koontz M., Seo K., Horlbeck M.A. (2020). CRISPRi-based radiation modifier screen identifies long non-coding RNA therapeutic targets in glioma. Genome Biol..

[90] Huang W., Shi Y., Han B., Wang Q., Zhang B., Qi C., Liu F. (2020). lncRNA GAS5-AS1 inhibits glioma proliferation, migration, and invasion via miR-106b-5p/TUSC2 axis. Hum. Cell.

[91] Liu L., Shi Y., Shi J., Wang H., Sheng Y., Jiang Q., Chen H., Li X., Dong J. (2019). The long non-coding RNA SNHG1 promotes glioma progression by competitively binding to miR-194 to regulate PHLDA1 expression. Cell Death Dis..

[92] Sheng J., He X., Yu W., Chen Y., Long Y., Wang K., Zhu S., Liu Q. (2021). p53-targeted lncRNA ST7-AS1 acts as a tumour suppressor by interacting with PTBP1 to suppress the Wnt/beta-catenin signalling pathway in glioma. Cancer Lett..

[93] Xiao M., Feng Y., Liu C., Zhang Z. (2018). Prognostic values of long noncoding RNA PVT1 in various carcinomas: An updated systematic review and meta-analysis. Cell Prolif..

[94] Xue W., Chen J., Liu X., Gong W., Zheng J., Guo X., Liu Y., Liu L., Ma J., Wang P. (2018). PVT1 regulates the malignant behaviors of human glioma cells by targeting miR-190a-5p and miR-488-3p. Biochim. Biophys. Acta Mol. Basis Dis..

[95] Qian Y., Shi L., Luo Z. (2020). Long Non-coding RNAs in Cancer: Implications for Diagnosis, Prognosis, and Therapy. Front. Med..

[96] Fu D., Shi Y., Liu J.B., Wu T.M., Jia C.Y., Yang H.Q., Zhang D.D., Yang X.L., Wang H.M., Ma Y.S. (2020). Targeting Long Non-coding RNA to Therapeutically Regulate Gene Expression in Cancer. Mol. Nucleic Acids.

[97] Anderson N.M., Mucka P., Kern J.G., Feng H. (2018). The emerging role and targetability of the TCA cycle in cancer metabolism. Protein Cell.

[98] Strickland M., Stoll E.A. (2017). Metabolic Reprogramming in Glioma. Front. Cell Dev. Biol..

[99] Labak C.M., Wang P.Y., Arora R., Guda M.R., Asuthkar S., Tsung A.J., Velpula K.K. (2016). Glucose transport: Meeting the metabolic demands of cancer, and applications in glioblastoma treatment. Am. J. Cancer Res..

[100] Lu W., Cao F., Wang S., Sheng X., Ma J. (2019). lncRNAs: The Regulator of Glucose and Lipid Metabolism in Tumor Cells. Front. Oncol..

[101] Lu J., Liu X., Zheng J., Song J., Liu Y., Ruan X., Shen S., Shao L., Yang C., Wang D. (2020). Lin28A promotes IRF6-regulated aerobic glycolysis in glioma cells by stabilizing SNHG14. Cell Death Dis..

[102] Boado R.J., Black K.L., Pardridge W.M. (1994). Gene expression of GLUT3 and GLUT1 glucose transporters in human brain tumors. Brain Res. Mol. Brain Res..

[103] Heydarzadeh S., Moshtaghie A.A., Daneshpoor M., Hedayati M. (2020). Regulators of glucose uptake in thyroid cancer cell lines. Cell Commun. Signal..

[104] Anderson M., Marayati R., Moffitt R., Yeh J.J. (2017). Hexokinase 2 promotes tumor growth and metastasis by regulating lactate production in pancreatic cancer. Oncotarget.

[105] Ciscato F., Ferrone L., Masgras I., Laquatra C., Rasola A. (2021). Hexokinase 2 in Cancer: A Prima Donna Playing Multiple Characters. Int. J. Mol. Sci..

[106] Sheikh T., Gupta P., Gowda P., Patrick S., Sen E. (2018). Hexokinase 2 and nuclear factor erythroid 2-related factor 2 transcriptionally coactivate xanthine oxidoreductase expression in stressed glioma cells. J. Biol. Chem..

[107] Zheng D., Che D., Lin F., Wang X., Lu L., Chen J., Xu X. (2020). lncRNA MACC1-AS1/MACC1 enhances the progression of glioma via regulating metabolic plasticity. Cell Cycle.

[108] Mukherjee J., Phillips J.J., Zheng S., Wiencke J., Ronen S.M., Pieper R.O. (2013). Pyruvate kinase M2 expression, but not pyruvate kinase activity, is up-regulated in a grade-specific manner in human glioma. PLoS ONE.

[109] Valvona C.J., Fillmore H.L., Nunn P.B., Pilkington G.J. (2016). The Regulation and Function of Lactate Dehydrogenase A: Therapeutic Potential in Brain Tumor. Brain Pathol..

[110] Mirzaei H., Hamblin M.R. (2020). Regulation of Glycolysis by Non-coding RNAs in Cancer: Switching on the Warburg Effect. Mol. Oncolytics.

[111] Liu R., Wang X., Shen Y., He A. (2021). Long non-coding RNA-based glycolysis-targeted cancer therapy: Feasibility, progression and limitations. Mol. Biol. Rep..

[112] Wang C., Li Y., Yan S., Wang H., Shao X., Xiao M., Yang B., Qin G., Kong R., Chen R. (2020). Interactome analysis reveals that lncRNA HULC promotes aerobic glycolysis through LDHA and PKM2. Nat. Commun..

[113] Bian Z., Zhang J., Li M., Feng Y., Wang X., Zhang J., Yao S., Jin G., Du J., Han W. (2018). lncRNA-FEZF1-AS1 Promotes Tumor Proliferation and Metastasis in Colorectal Cancer by Regulating PKM2 Signaling. Clin. Cancer Res..

[114] Liu X., Zhu Q., Guo Y., Xiao Z., Hu L., Xu Q. (2019). lncRNA LINC00689 promotes the growth, metastasis and glycolysis of glioma cells by targeting miR-338-3p/PKM2 axis. Biomed. Pharmacol..

[115] Abdel-Magid A.F. (2020). Inhibitors of Hypoxia-Inducible Factors as Treatment for Cancer. ACS Med. Chem. Lett..

[116] Ziello J.E., Jovin I.S., Huang Y. (2007). Hypoxia-Inducible Factor (HIF)-1 regulatory pathway and its potential for therapeutic intervention in malignancy and ischemia. Yale J. Biol. Med..

[117] Nagao A., Kobayashi M., Koyasu S., Chow C.C.T., Harada H. (2019). HIF-1-Dependent Reprogramming of Glucose Metabolic Pathway of Cancer Cells and Its Therapeutic Significance. Int. J. Mol. Sci..

[118] Kierans S.J., Taylor C.T. (2021). Regulation of glycolysis by the hypoxia-inducible factor (HIF): Implications for cellular physiology. J. Physiol..

[119] Yao Z., Zhang Q., Guo F., Guo S., Yang B., Liu B., Li P., Li J., Guan S., Liu X. (2020). Long Noncoding RNA PCED1B-AS1 Promotes the Warburg Effect and Tumorigenesis by Upregulating HIF-1alpha in Glioblastoma. Cell Transpl..

[120] Barth D.A., Prinz F., Teppan J., Jonas K., Klec C., Pichler M. (2020). Long-Noncoding RNA (lncRNA) in the Regulation of Hypoxia-Inducible Factor (HIF) in Cancer. Noncoding RNA.

[121] Choudhry H., Mole D.R. (2016). Hypoxic regulation of the noncoding genome and NEAT1. Brief. Funct. Genom..

[122] Wang X., Zhao D., Xie H., Hu Y. (2021). Interplay of long non-coding RNAs and HIF-1alpha: A new dimension to understanding hypoxia-regulated tumor growth and metastasis. Cancer Lett..

[123] Xiao Y., Peng H., Hong C., Chen Z., Deng X., Wang A., Yang F., Yang L., Chen C., Qin X. (2017). PDGF Promotes the Warburg Effect in Pulmonary Arterial Smooth Muscle Cells via Activation of the PI3K/AKT/mTOR/HIF-1alpha Signaling Pathway. Cell Physiol. Biochem..

[124] Jean S., Kiger A.A. (2014). Classes of phosphoinositide 3-kinases at a glance. J. Cell Sci..

[125] Fattahi S., Amjadi-Moheb F., Tabaripour R., Ashrafi G.H., Akhavan-Niaki H. (2020). PI3K/AKT/mTOR signaling in gastric cancer: Epigenetics and beyond. Life Sci..

[126] Wang Y., Kuramitsu Y., Baron B., Kitagawa T., Tokuda K., Akada J., Maehara S.I., Maehara Y., Nakamura K. (2017). PI3K inhibitor LY294002, as opposed to wortmannin, enhances AKT phosphorylation in gemcitabine-resistant pancreatic cancer cells. Int. J. Oncol..

[127] Yu L., Chen X., Sun X., Wang L., Chen S. (2017). The Glycolytic Switch in Tumors: How Many Players Are Involved?. J. Cancer.

[128] Feng J., Li J., Wu L., Yu Q., Ji J., Wu J., Dai W., Guo C. (2020). Emerging roles and the regulation of aerobic glycolysis in hepatocellular carcinoma. J. Exp. Clin. Cancer Res..

[129] Aboudehen K. (2020). Regulation of mTOR signaling by long non-coding RNA. Biochim. Biophys. Acta Gene Regul. Mech..

[130] Liu C.H., Zhang Y., She X.L., Fan L., Li P.Y., Feng J.B., Fu H.J., Liu Q., Liu Q., Zhao C.H. (2018). A cytoplasmic long noncoding RNA LINC00470 as a new AKT activator to mediate glioblastoma cell autophagy. J. Hematol. Oncol..

[131] Cheng Z., Luo C., Guo Z. (2020). lncRNA-XIST/microRNA-126 sponge mediates cell proliferation and glucose metabolism through the IRS1/PI3K/Akt pathway in glioma. J. Cell Biochem..

[132] Liu R., Chen Y.W., Liu G.Z., Li C.X., Song Y.R., Cao Z.W., Li W., Hu J.H., Lu C., Liu Y.Y. (2020). PI3K/AKT pathway as a key link modulates the multidrug resistance of cancers. Cell Death Dis..

[133] Caspi M., Wittenstein A., Kazelnik M., Shor-Nareznoy Y., Rosin-Arbesfeld R. (2021). Therapeutic targeting of the oncogenic Wnt signaling pathway for treating colorectal cancer and other colonic disorders. Adv. Drug Deliv. Rev..

[134] Martin-Orozco E., Sanchez-Fernandez A., Ortiz-Parra I., Ayala-San Nicolas M. (2019). WNT Signaling in Tumors: The Way to Evade Drugs and Immunity. Front. Immunol..

[135] Sethi J.K., Vidal-Puig A. (2010). Wnt signalling and the control of cellular metabolism. Biochem. J..

[136] Mo Y., Wang Y., Zhang L., Yang L., Zhou M., Li X., Li Y., Li G., Zeng Z., Xiong W. (2019). The role of Wnt signaling pathway in tumor metabolic reprogramming. J. Cancer.

[137] Sherwood V. (2015). WNT signaling: An emerging mediator of cancer cell metabolism?. Mol. Cell Biol..

[138] Zhang H., Qin D., Jiang Z., Zhang J. (2019). SNHG9/miR-199a-5p/Wnt2 Axis Regulates Cell Growth and Aerobic Glycolysis in Glioblastoma. J. Neuropathol. Exp. Neurol..

[139] Guo H., Hu G., Yang Q., Zhang P., Kuang W., Zhu X., Wu L. (2016). Knockdown of long non-coding RNA CCAT2 suppressed proliferation and migration of glioma cells. Oncotarget.

[140] Li Y.Q., Zhu G., Zeng W., Wang J.C., Li Z.H., Wang B., Tian B., Lu D., Zhang X.Y., Gao G.D. (2017). Long noncoding RNA AB073614 promotes the malignance of glioma by activating Wnt/beta-catenin signaling through downregulating SOX7. Oncotarget.

[141] Zhang H., Wei D.L., Wan L., Yan S.F., Sun Y.H. (2017). Highly expressed lncRNA CCND2-AS1 promotes glioma cell proliferation through Wnt/beta-catenin signaling. Biochem. Biophys. Res. Commun..

[142] Li J., Zhou L. (2018). Overexpression of lncRNA DANCR positively affects progression of glioma via activating Wnt/beta-catenin signaling. Biomed. Pharmacol..

[143] El-Sahli S., Xie Y., Wang L., Liu S. (2019). Wnt Signaling in Cancer Metabolism and Immunity. Cancers.

[144] Wang Y., Zhang Y.R., Cai G., Li Q. (2020). Exosomes as Actively Targeted Nanocarriers for Cancer Therapy. Int. J. Nanomed..

